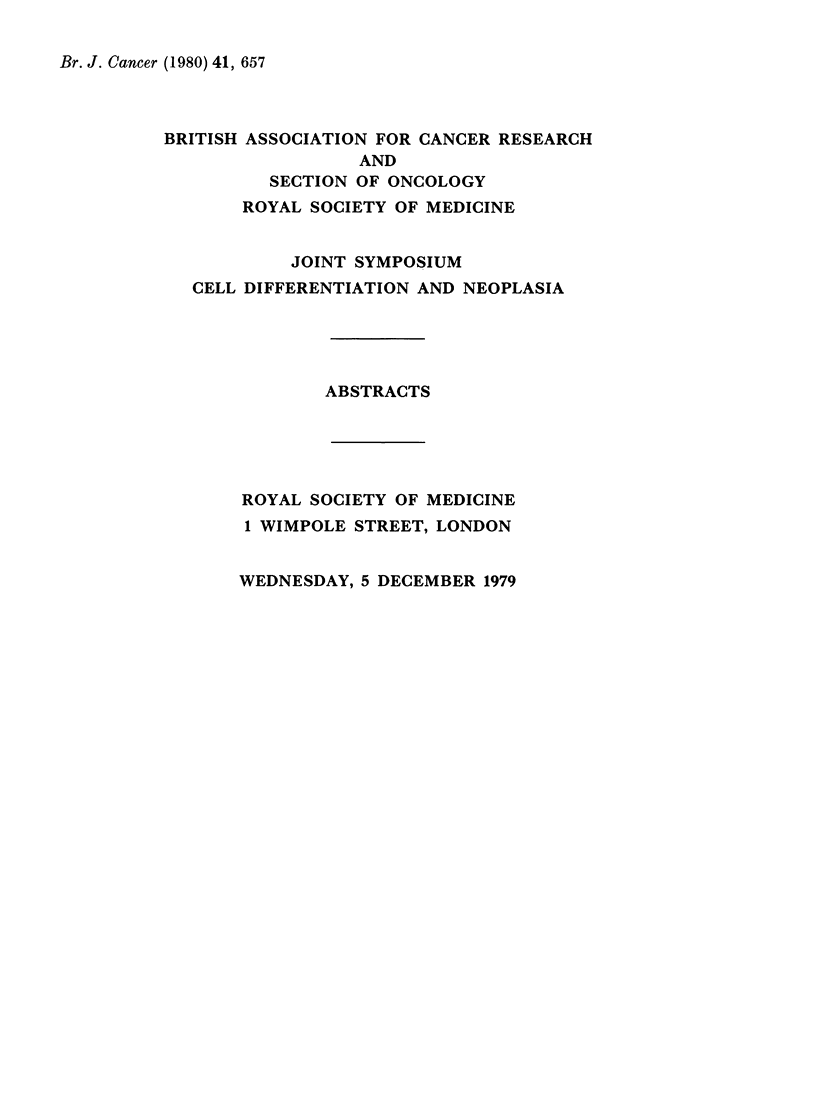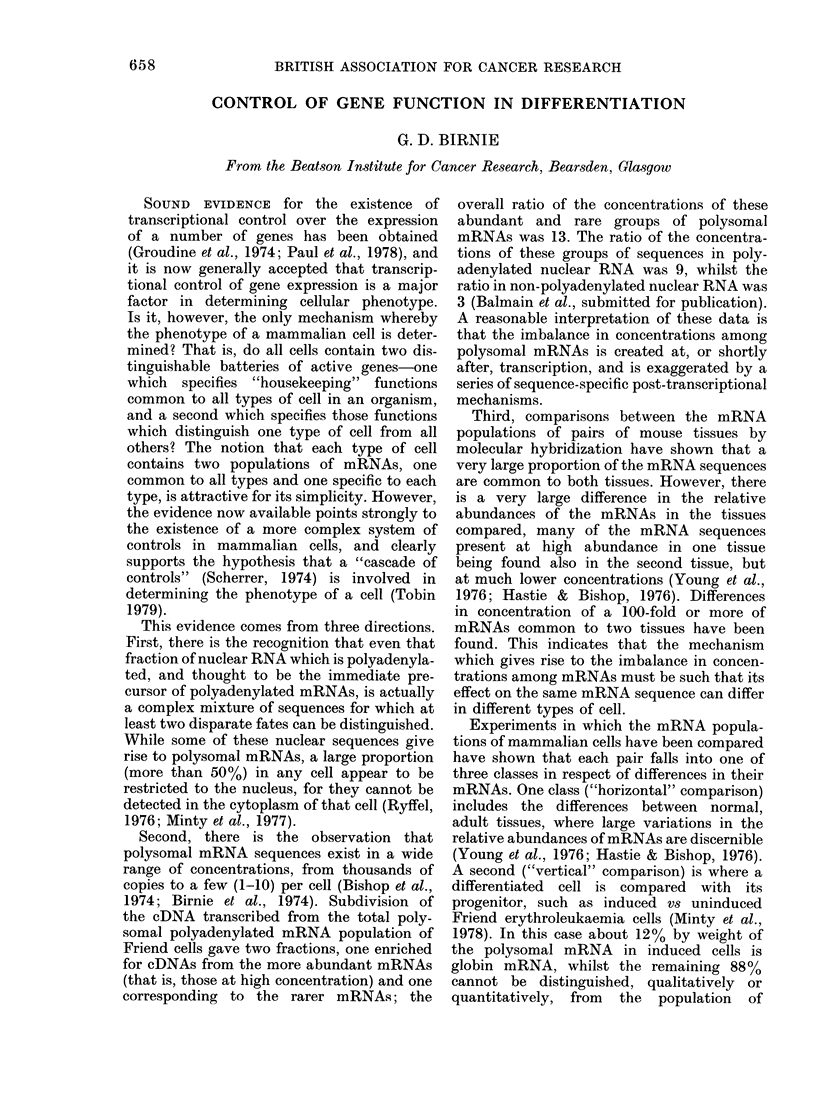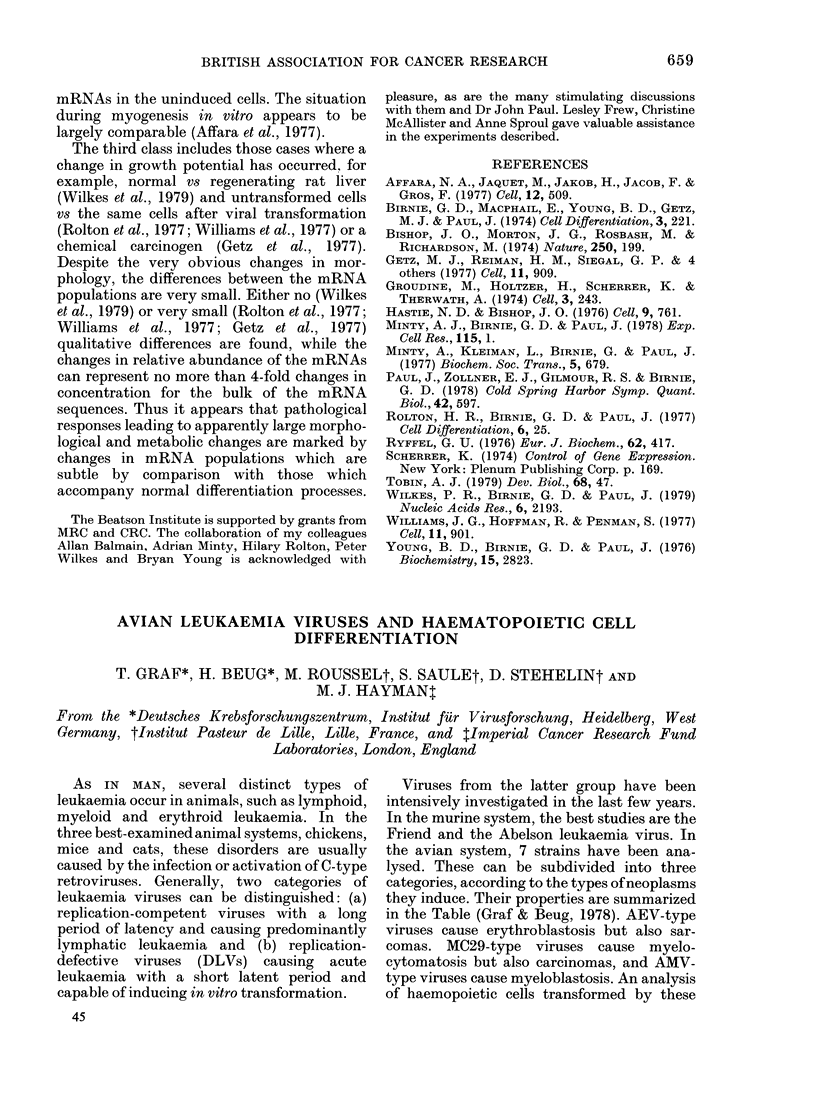# Control of gene function in differentiation.

**DOI:** 10.1038/bjc.1980.113

**Published:** 1980-04

**Authors:** G. D. Birnie


*Br. J. Cancer* (1980) **41**, 657

### BRITISH ASSOCIATION FOR CANCER RESEARCH
### AND
### SECTION OF ONCOLOGY
### ROYAL SOCIETY OF MEDICINE

### JOINT SYMPOSIUM
### CELL DIFFERENTIATION AND NEOPLASIA

---

### ABSTRACTS

---

ROYAL SOCIETY OF MEDICINE
1 WIMPOLE STREET, LONDON

WEDNESDAY, 5 DECEMBER 1979

### CONTROL OF GENE FUNCTION IN DIFFERENTIATION

G. D. BIRNIE

*From the Beatson Institute for Cancer Research, Bearsden, Glasgow*

SOUND EVIDENCE for the existence of transcriptional control over the expression of a number of genes has been obtained (Groudine *et al.*, 1974; Paul *et al.*, 1978), and it is now generally accepted that transcriptional control of gene expression is a major factor in determining cellular phenotype. Is it, however, the only mechanism whereby the phenotype of a mammalian cell is determined? That is, do all cells contain two distinguishable batteries of active genes—one which specifies "housekeeping" functions common to all types of cell in an organism, and a second which specifies those functions which distinguish one type of cell from all others? The notion that each type of cell contains two populations of mRNAs, one common to all types and one specific to each type, is attractive for its simplicity. However, the evidence now available points strongly to the existence of a more complex system of controls in mammalian cells, and clearly supports the hypothesis that a "cascade of controls" (Scherrer, 1974) is involved in determining the phenotype of a cell (Tobin 1979).

This evidence comes from three directions. First, there is the recognition that even that fraction of nuclear RNA which is polyadenylated, and thought to be the immediate precursor of polyadenylated mRNAs, is actually a complex mixture of sequences for which at least two disparate fates can be distinguished. While some of these nuclear sequences give rise to polysomal mRNAs, a large proportion (more than 50%) in any cell appear to be restricted to the nucleus, for they cannot be detected in the cytoplasm of that cell (Ryffel, 1976; Minty *et al.*, 1977).

Second, there is the observation that polysomal mRNA sequences exist in a wide range of concentrations, from thousands of copies to a few (1–10) per cell (Bishop *et al.*, 1974; Birnie *et al.*, 1974). Subdivision of the cDNA transcribed from the total polysomal polyadenylated mRNA population of Friend cells gave two fractions, one enriched for cDNAs from the more abundant mRNAs (that is, those at high concentration) and one corresponding to the rarer mRNAs; the overall ratio of the concentrations of these abundant and rare groups of polysomal mRNAs was 13. The ratio of the concentrations of these groups of sequences in polyadenylated nuclear RNA was 9, whilst the ratio in non-polyadenylated nuclear RNA was 3 (Balmain *et al.*, submitted for publication). A reasonable interpretation of these data is that the imbalance in concentrations among polysomal mRNAs is created at, or shortly after, transcription, and is exaggerated by a series of sequence-specific post-transcriptional mechanisms.

Third, comparisons between the mRNA populations of pairs of mouse tissues by molecular hybridization have shown that a very large proportion of the mRNA sequences are common to both tissues. However, there is a very large difference in the relative abundances of the mRNAs in the tissues compared, many of the mRNA sequences present at high abundance in one tissue being found also in the second tissue, but at much lower concentrations (Young *et al.*, 1976; Hastie & Bishop, 1976). Differences in concentration of a 100-fold or more of mRNAs common to two tissues have been found. This indicates that the mechanism which gives rise to the imbalance in concentrations among mRNAs must be such that its effect on the same mRNA sequence can differ in different types of cell.

Experiments in which the mRNA populations of mammalian cells have been compared have shown that each pair falls into one of three classes in respect of differences in their mRNAs. One class ("horizontal" comparison) includes the differences between normal, adult tissues, where large variations in the relative abundances of mRNAs are discernible (Young *et al.*, 1976; Hastie & Bishop, 1976). A second ("vertical" comparison) is where a differentiated cell is compared with its progenitor, such as induced *vs* uninduced Friend erythroleukaemia cells (Minty *et al.*, 1978). In this case about 12% by weight of the polysomal mRNA in induced cells is globin mRNA, whilst the remaining 88% cannot be distinguished, qualitatively or quantitatively, from the population of mRNAs in the uninduced cells. The situation during myogenesis *in vitro* appears to be largely comparable (Affara *et al.*, 1977).

The third class includes those cases where a change in growth potential has occurred, for example, normal *vs* regenerating rat liver (Wilkes *et al.*, 1979) and untransformed cells *vs* the same cells after viral transformation (Rolton *et al.*, 1977; Williams *et al.*, 1977) or a chemical carcinogen (Getz *et al.*, 1977). Despite the very obvious changes in morphology, the differences between the mRNA populations are very small. Either no (Wilkes *et al.*, 1979) or very small (Rolton *et al.*, 1977; Williams *et al.*, 1977; Getz *et al.*, 1977) qualitative differences are found, while the changes in relative abundance of the mRNAs can represent no more than 4-fold changes in concentration for the bulk of the mRNA sequences. Thus it appears that pathological responses leading to apparently large morphological and metabolic changes are marked by changes in mRNA populations which are subtle by comparison with those which accompany normal differentiation processes.

The Beatson Institute is supported by grants from MRC and CRC. The collaboration of my colleagues Allan Balmain, Adrian Minty, Hilary Rolton, Peter Wilkes and Bryan Young is acknowledged with pleasure, as are the many stimulating discussions with them and Dr John Paul. Lesley Frew, Christine McAllister and Anne Sproul gave valuable assistance in the experiments described.

REFERENCES

Affara, N. A., Jaquet, M., Jakob, H., Jacob, F. & Gros, F. (1977) *Cell*, **12**, 509.
Birnie, G. D., Macphail, E., Young, B. D., Getz, M. J. & Paul, J. (1974) *Cell Differentiation*, **3**, 221.
Bishop, J. O., Morton, J. G., Rosbash, M. & Richardson, M. (1974) *Nature*, **250**, 199.
Getz, M. J., Reiman, H. M., Siegal, G. P. & 4 others (1977) *Cell*, **11**, 909.
Groudine, M., Holtzer, H., Scherrer, K. & Therwath, A. (1974) *Cell*, **3**, 243.
Hastie, N. D. & Bishop, J. O. (1976) *Cell*, **9**, 761.
Minty, A. J., Birnie, G. D. & Paul, J. (1978) *Exp. Cell Res.*, **115**, 1.
Minty, A., Kleiman, L., Birnie, G. & Paul, J. (1977) *Biochem. Soc. Trans.*, **5**, 679.
Paul, J., Zollner, E. J., Gilmour, R. S. & Birnie, G. D. (1978) *Cold Spring Harbor Symp. Quant. Biol.*, **42**, 597.
Rolton, H. R., Birnie, G. D. & Paul, J. (1977) *Cell Differentiation*, **6**, 25.
Ryffel, G. U. (1976) *Eur. J. Biochem.*, **62**, 417.
Scherrer, K. (1974) *Control of Gene Expression*. New York: Plenum Publishing Corp. p. 169.
Tobin, A. J. (1979) *Dev. Biol.*, **68**, 47.
Wilkes, P. R., Birnie, G. D. & Paul, J. (1979) *Nucleic Acids Res.*, **6**, 2193.
Williams, J. G., Hoffman, R. & Penman, S. (1977) *Cell*, **11**, 901.
Young, B. D., Birnie, G. D. & Paul, J. (1976) *Biochemistry*, **15**, 2823.